# Prevention of Prescription Opioid Misuse and Projected Overdose Deaths in the United States

**DOI:** 10.1001/jamanetworkopen.2018.7621

**Published:** 2019-02-01

**Authors:** Qiushi Chen, Marc R. Larochelle, Davis T. Weaver, Anna P. Lietz, Peter P. Mueller, Sarah Mercaldo, Sarah E. Wakeman, Kenneth A. Freedberg, Tiana J. Raphel, Amy B. Knudsen, Pari V. Pandharipande, Jagpreet Chhatwal

**Affiliations:** 1 Harold and Inge Marcus Department of Industrial and Manufacturing Engineering, Pennsylvania State University, University Park, Pennsylvania; 2 Institute for Technology Assessment, Department of Radiology, Massachusetts General Hospital, Boston; 3 Harvard Medical School, Boston, Massachusetts; 4 Clinical Addiction Research and Education Unit, Section of General Internal Medicine, Department of Medicine, Boston University School of Medicine and Boston Medical Center, Boston, Massachusetts; 5 School of Medicine, Case Western Reserve University, Cleveland, Ohio; 6 Division of General Internal Medicine, Department of Medicine, Massachusetts General Hospital, Boston; 7 Medical Practice Evaluation Center, Massachusetts General Hospital, Boston; 8 Department of Health Policy and Management, Harvard T.H. Chan School of Public Health, Boston, Massachusetts; 9 University of Texas Southwestern Medical School, Dallas

## Abstract

**Importance:**

Deaths due to opioid overdose have tripled in the last decade. Efforts to curb this trend have focused on restricting the prescription opioid supply; however, the near-term effects of such efforts are unknown.

**Objective:**

To project effects of interventions to lower prescription opioid misuse on opioid overdose deaths from 2016 to 2025.

**Design, Setting, and Participants:**

This system dynamics (mathematical) model of the US opioid epidemic projected outcomes of simulated individuals who engage in nonmedical prescription or illicit opioid use from 2016 to 2025. The analysis was performed in 2018 by retrospectively calibrating the model from 2002 to 2015 data from the National Survey on Drug Use and Health and the Centers for Disease Control and Prevention.

**Interventions:**

Comparison of interventions that would lower the incidence of prescription opioid misuse from 2016 to 2025 based on historical trends (a 7.5% reduction per year) and 50% faster than historical trends (an 11.3% reduction per year), vs a circumstance in which the incidence of misuse remained constant after 2015.

**Main Outcomes and Measures:**

Opioid overdose deaths from prescription and illicit opioids from 2016 to 2025 under each intervention.

**Results:**

Under the status quo, the annual number of opioid overdose deaths is projected to increase from 33 100 in 2015 to 81 700 (95% uncertainty interval [UI], 63 600-101 700) in 2025 (a 147% increase from 2015). From 2016 to 2025, 700 400 (95% UI, 590 200-817 100) individuals in the United States are projected to die from opioid overdose, with 80% of the deaths attributable to illicit opioids. The number of individuals using illicit opioids is projected to increase by 61%—from 0.93 million (95% UI, 0.83-1.03 million) in 2015 to 1.50 million (95% UI, 0.98-2.22 million) by 2025. Across all interventions tested, further lowering the incidence of prescription opioid misuse from 2015 levels is projected to decrease overdose deaths by only 3.0% to 5.3%.

**Conclusions and Relevance:**

This study’s findings suggest that interventions targeting prescription opioid misuse such as prescription monitoring programs may have a modest effect, at best, on the number of opioid overdose deaths in the near future. Additional policy interventions are urgently needed to change the course of the epidemic.

## Key Points

**Question:**

What is the projected effect of lowering incident nonmedical prescription opioid use on the future trajectory of the opioid overdose crisis in the United States?

**Findings:**

In this system dynamics model study, under current conditions, the opioid overdose crisis is expected to worsen—with the annual number of opioid overdose deaths projected to reach nearly 82 000 by 2025, resulting in approximately 700 000 deaths from 2016 to 2025. Interventions focused on lowering the incidence of prescription opioid misuse were projected to result in a 3.0% to 5.3% decrease in opioid overdose deaths over this period.

**Meaning:**

Prevention of prescription opioid misuse alone is projected to have a modest effect on lowering opioid overdose deaths in the near future, and multipronged approach is needed to dramatically change the course of the epidemic.

## Teaser

This system dynamics (mathematical) model projects effects of interventions to lower prescription opioid misuse on opioid overdose deaths from 2016 to 2025.

## Introduction

In the last decade, US deaths due to opioid-related overdoses have tripled, increasing from approximately 17 500 in 2006 to 42 200 in 2016.^1^ In October 2017, the US Department of Health and Human Services declared the opioid crisis a national public health emergency.^2^ The opioid crisis has also resulted in a substantial cost burden to society—in 2013, health care costs, criminal justice expenses, and productivity losses attributable to opioid misuse were estimated to total $78.5 billion; this cost is expected to increase further in the coming years.^3^

To date, efforts to curb the course of the opioid overdose epidemic have principally focused on restricting the supply of prescription opioid analgesics through prescription drug monitoring programs, opioid prescribing guidelines,^4^ dose-limit laws, prescription drug take-back days, and law enforcement approaches.^5,6,7,8,9,10,11^ One premise of these supply-side interventions is that they will decrease the number of individuals exposed to opioid analgesics and subsequently prevent individuals from developing an opioid use disorder (OUD), ultimately lowering the number of opioid overdose deaths. Previous studies have shown that such interventions may lead to a modest decrease in the prescription opioid supply, especially programs with enrollment and/or use mandates.^5,6,7,10,12,13,14^ However, analyses of these programs have failed to demonstrate a consistent benefit on fatal or nonfatal opioid overdoses.^15^

Furthermore, the nature of the opioid epidemic has shifted in recent years. Many people who previously may have misused prescription opioids now use illicit opioids such as heroin and fentanyl (a synthetic opioid),^16^ and an increasing number of people initiate opioid use with illicit rather than prescription opioids.^17^ As such, the effect of interventions focused on lowering the prescription opioid supply and prescription opioid misuse on the future trajectory of the opioid epidemic is less clear.^18,19,20^

Our goal was to project the effect of efforts to prevent the misuse of prescription opioid analgesics on future overdose deaths. To accomplish this goal, we simulated the changing trajectory of the opioid overdose crisis over time and evaluated the potential effect of lowering the incidence of prescription opioid misuse on overdose deaths in the United States through 2025.

## Methods

### Overview

Using data from the National Survey on Drug Use and Health (NSDUH),^21^ the Centers for Disease Control and Prevention (CDC),^1^ and published data,^17^ we developed a mathematical model, the Opioid Policy Model*,* to simulate the opioid overdose crisis in the United States from 2002 to 2025. We calibrated the model to reproduce observed trends of opioid misuse and opioid overdose deaths up to year 2015, and then used it to project these outcomes from 2016 to 2025. Finally, we evaluated the effects of lowering the incidence of nonmedical opioid analgesic use on the projected number of opioid overdose deaths. All data used in this study were publicly available and therefore did not require approval from an institutional review board. We followed the Consolidated Health Economic Evaluation Reporting Standards (CHEERS) reporting guideline for reporting our study design and outcomes.^22^

### Opioid Policy Model

**Figure 1.**
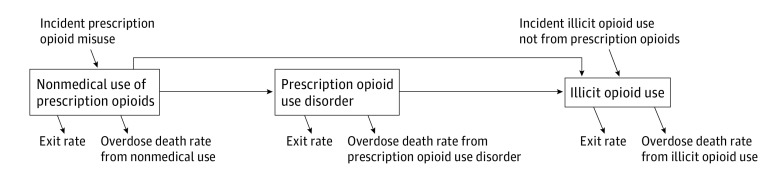
Overview of the System Dynamics Model of Nonmedical Opioid Use Persons using opioids nonmedically are represented in the model in 1 of 3 compartments: nonmedical use of prescription opioids without opioid use disorder, prescription opioid use disorder, and illicit opioid use. New individuals can enter the model using prescription opioids or illicit opioids and transition through different states of opioid use (arrows). Individuals can die from opioid overdose with mortality rates dependent on their compartment or can transition out of the model when they either stop using opioids or die from other (ie, nonopioid-related) causes. We assumed that prevention of prescription opioid misuse will lower the incidence of prescription opioid misuse and evaluated their effect on overdose deaths.

We developed a system dynamics model, also known as a compartment model,^23^ to simulate the trajectory of nonmedical opioid use in the United States starting from the year 2002. The model consists of 3 compartments that distinguish 3 subgroups of the population using opioids nonmedically (Figure 1): those using prescription opioids nonmedically without an OUD; those with a prescription OUD; and those using illicit opioids (with and without the diagnosis of OUD, possibly with simultaneous use of prescription opioids) (eAppendix 1 and eFigure 1 in the Supplement).

New individuals can enter the model using either prescription opioids nonmedically without an OUD or illicit opioids. Individuals with nonmedical use of prescription opioids without an OUD can develop a prescription OUD. Individuals who use prescription opioids, with or without prescription OUD, can transition to illicit opioid use, defined by initiating use of heroin or fentanyl. Individuals can die from opioid overdose with mortality rates dependent on their compartment. In addition, individuals can transition out of the model when they either stop using opioids or die from other (ie, nonopioid-related) causes. Model programming and analysis were performed in R, version 3.4.0 (R Foundation for Statistical Computing).

### Data Sources

We used 2 major national data sources for model development: the NSDUH and multiple cause of death data from the CDC’s Wide-Ranging Online Data for Epidemiologic Research. The NSDUH provides data on the prevalence and incidence of drug use in the United States.^21^ We used data from 2002 to 2015, which includes more than 55 000 respondents each year. We generated estimates of the annual prevalence of nonmedical use of prescription opioids without an OUD, prescription OUD (as defined by the criteria of *Diagnostic and Statistical Manual of Mental Disorders, Fourth Edition* for opioid abuse or dependence), and illicit opioid use (ie, heroin and fentanyl), and the annual incidence of prescription opioid misuse. The estimates were adjusted by survey weights that account for the complex sampling design of the NSDUH.^24^

We used the CDC’s Wide-Ranging Online Data for Epidemiologic Research multiple cause of death data to estimate the numbers of annual overdose deaths associated with prescription and illicit opioid use from 2002 to 2015. We used underlying cause of death *International Statistical Classification of Diseases, Tenth Revision* codes (X40-X44, X61-X64, X85,Y10-Y14) and multiple cause of death *International Statistical Classification of Diseases, Tenth Revision* codes (T40.0-T40.4, T40.6) to identify all deaths related to overdose from opioid use, where deaths related to heroin use (T40.1) and fentanyl (part of other synthetic opioids coded as T40.4) were grouped as overdose deaths from illicit opioid use.

To account for the recent, rapid increase in individuals who initiate opioid use with heroin, not prescription opioids, we used published estimates on the proportion of individuals who reported initiating opioid use with heroin from 2005 to 2015.^17^

### Model Parameter Estimation and Calibration

We estimated the initial distribution of the population using opioids nonmedically in the starting year 2002 and the annual incidence of nonmedical use of prescription opioids from 2002 to 2015 from the NSDUH data (eAppendix 1 in the Supplement). Because some model parameters like transition rates between compartments may not be constant over time, we conducted joinpoint regression analysis to inform the time-dependent structures for these model parameters (eAppendix 1, eFigure 2, and eTable 1 in the Supplement). For model parameters that could not be directly estimated from available data, we inferred their values via calibration to match model outcomes with the data that are observable (eAppendix 2 in the Supplement). In particular, we calibrated the transition rates between compartments, exit rates and overdose mortality rates from each compartment, and the incidence of illicit opioid use. Observable data, used as calibration targets, included the annual prevalence for each compartment for 2002 through 2015 from NSDUH,^21^ the annual number of overdose deaths from all opioids and from illicit opioids only for 2002 through 2015 from the CDC Wide-Ranging Online Data for Epidemiologic Research,^1^ and the percentage of individuals who initiate opioid use with illicit (rather than prescription) opioids from 2005 to 2015 from the published literature (eTables 2 and 3 in the Supplement).^17^ We applied a directed search algorithm^25^ to identify the values of calibrated parameters such that model outcomes closely matched observable data between 2002 and 2015. To account for uncertainty in the calibrated parameter values, we repeated the calibration process 1000 times, resulting in 1000 independent sets of calibrated model parameters (eFigure 3 and eTable 4 in the Supplement).

### Assumptions of Lethality and Incidence of Illicit Opioid Use

Because of the highly dynamic nature of the opioid overdose crisis, it is unclear how the overdose mortality rate from illicit opioids and the rate of incident illicit opioid use will continue to increase over time in the future. In the base case, we assumed that the lethality and rate of incident illicit opioid use would continue to increase after 2015. This assumption was made to account for the possibility of an increasing rate of overdose deaths from illicit opioid use, driven by continued infiltration of highly potent synthetic opioids such as fentanyl and carfentanil^26,27^ and the increasing incidence rate of illicit opioids as the first opioid of use, as observed in the preceding years.^1,17^ However, because these rates are unlikely to continue to increase indefinitely, we assumed that the increasing trends of both rates would stabilize by 2020 (eAppendix 3 and eFigure 4 in the Supplement). This assumption was based on data from Massachusetts that showed saturation in the use of fentanyl and stabilization of the overdose rate in the state in 2017,^28^ implying that such stabilization could happen in other states in the near future. To consider the possibility that the crisis may continue to worsen for a longer period, in our sensitivity analysis, we also evaluated a pessimistic scenario, that assumed the increasing rates would stabilize in the year 2025.

### Simulated Strategies to Reduce the Incidence of Nonmedical Prescription Opioid Use

We evaluated the projected effect of lowering the incidence of nonmedical prescription opioid use on the opioid overdose deaths from 2016 to 2025. In particular, we simulated the following prevention strategies (eAppendix 3 and eFigure 5 in the Supplement): no change in the annual incidence of prescription opioid misuse from 2015 onward (reference case); decreasing incidence of prescription opioid misuse by 7.5% per year from 2016 to 2025, based on the observed trends of the incidence estimates from the NSDUH between 2011 and 2015; decreasing incidence of prescription opioid misuse at a rate that is 50% faster than strategy 2 (ie, an 11.3% decrease per year); and a hypothetical circumstance of no new incidence of prescription opioid misuse after 2015, which was included to assess the maximum possible benefit of prevention interventions for prescription opioid misuse. Because it is difficult to measure the effects of a particular supply-side intervention on the decrease of the incidence of prescription opioid misuse, and because such reduction is a result of multiple factors, including the success of different programs preventing prescription opioid misuse, we evaluated outcomes over a wide range of effectiveness of such interventions.

### Outcomes

Primary outcomes were the annual number of overdose deaths from all opioids and from illicit opioids from 2016 to 2025, and the cumulative number of opioid overdose deaths during the same period. In addition, we projected temporal trends in the nonmedical use of prescription opioids without an OUD, prescription OUD, and illicit opioid use between 2016 and 2025.

### Statistical (Uncertainty) Analysis

To quantify the uncertainty in outcomes arising from the uncertainty in the calibrated parameter values, we repeated the analysis 1000 times (ie, once for each unique set of calibrated parameters). Results are presented as the mean value and the 95% uncertainty interval (UI) using the 2.5th to 97.5th percentile range across the 1000 evaluations. In addition, we conducted a sensitivity analysis to account for missing institutionalized populations in the NSDUH data (eAppendix 4 in the Supplement).

## Results

### Opioid Overdose Crisis Projections

**Figure 2.**
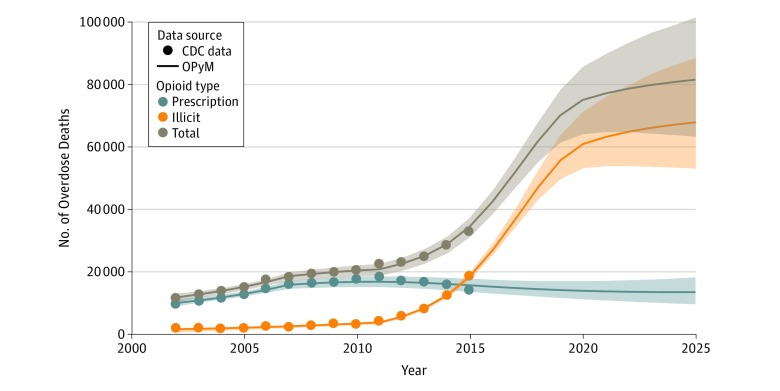
Overdose Deaths From Prescription and Illicit Opioids From 2002 to 2025 Under the Base-Case Projection Scenario The model closely replicated the overdose deaths reported by the Centers for Disease Control and Prevention (CDC) from 2002 to 2015 and projected that the number of overdose deaths will increase substantially from 2016 onward. The lines are the average outcomes across 1000 calibrated parameter sets. Shaded regions represent the bootstrapped 95% uncertainty intervals of the model outcomes. OPyM indicates opioid policy model.

We projected that under the status quo, the total number of opioid overdose deaths in the United States will increase from 33 100 in 2015 to 81 700 (95% UI, 63 600-101 700) by 2025 (a 147% increase) (Figure 2). The majority of these deaths would result from illicit opioid use—overdose deaths from illicit opioids are projected to increase from 18 900 in 2015 to 67 900 (95% UI, 52 200-86 700) in 2025 (a 259% increase). In contrast, overdose deaths from prescription opioid use (with or without OUD) would marginally decrease from 14 200 in 2015 to 13 800 (95% UI, 9900-18 500) in 2025 (a 3% decrease). We estimated that between 2016 and 2025, 700 400 (95% UI, 590 200-817 100) individuals will die from opioid overdose, with 80% (ie, 557 100; 95% UI, 466 400-658 200) of the deaths attributable to illicit opioids.

**Figure 3.**
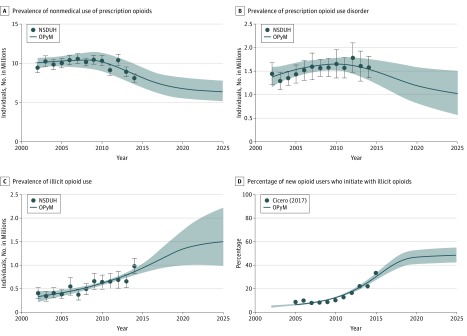
Temporal Trends in the Opioid Overdose Crisis for the Base-Case Scenario, 2002-2025 A, Prevalence of nonmedical use of prescription opioids. B, Prevalence of prescription opioid use disorder. C, Prevalence of illicit opioid use. D, Percentage of individuals who initiate opioid use with an illicit opioid (rather than a prescription opioid). The model was calibrated to closely replicate observed outcomes from 2002 to 2015 and used to project outcomes from 2015 to 2025. Lines represent the average of 1000 outcomes from the model. Error bars represent 95% confidence intervals of the observed outcomes from the National Survey on Drug Use and Health (NSDUH) data, and shaded regions represent the bootstrapped 95% uncertainty intervals of the model outcomes. Cicero (2017)^17^ indicates the source of calibration targets; and OPyM, opioid policy model.

The number of people using prescription opioids nonmedically without an OUD is projected to decrease from 8.15 million (95% UI, 6.84-9.41 million) people in 2015 to 6.36 million (95% UI, 5.13-7.79 million) by 2025 (a 22% decrease) (Figure 3A). Similarly, the prevalence of prescription OUD is projected to decrease by 31%—from 1.49 million individuals (95% UI, 1.22-1.74 million) in 2015 to 1.03 million individuals (95% UI, 0.57-1.51 million) in 2025 (Figure 3B). In contrast, the number of individuals using illicit opioids is projected to increase by 61%—from 0.93 million (95% UI, 0.83-1.03 million) in 2015 to 1.50 million (95% UI, 0.98-2.22 million) by 2025 (Figure 3C). During the same period, the percentage of people who initiate opioid use with an illicit opioid is projected to increase from 30% (95% UI, 27%-33%) to 48% (95% UI, 42%-55%) (Figure 3D).

### Projected Effect of Prevention Strategies

**Figure 4.**
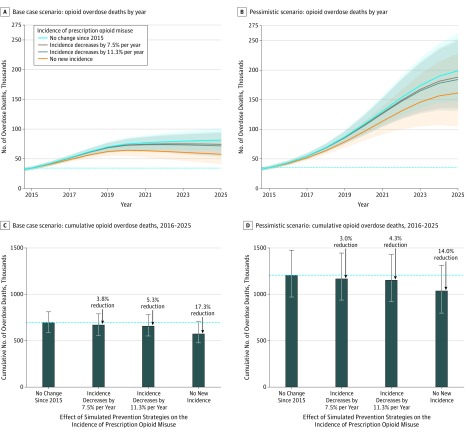
Projected Effects of Preventing New Cases of Prescription Opioid Misuse in the Base Case and Pessimistic Scenarios A and B, Projection of overdose deaths by year in the base case (A) and pessimistic (B) scenarios under 4 prevention strategies affecting the incidence of nonmedical opioid analgesic use: (1) no change in the annual incidence of prescription opioid misuse since 2015, (2) decreasing incidence of prescription opioid misuse at the rate observed between 2011 and 2015 (ie, 7.5% decrease per year), (3) decreasing incidence of prescription opioid misuse at a rate that is 50% faster than strategy 2, ie, 11.3% decrease per year, and (4) no new incidence after 2015. C and D, Cumulative overdose deaths by prevention strategy under the base case (C) and pessimistic (D) scenarios. The dotted lines indicate the reference values: the number of overdose deaths in 2015 (A and B), and the cumulative number of overdose deaths for scenario (1) (C and D); the shaded areas in A and B and the error bars in C and D indicate the 95% uncertainty interval of model outcomes. The base-case scenario assumed that the opioid overdose crisis will stabilize by 2020, ie, the incidence of illicit opioids as the initiating opioid and the overdose mortality rate attributable to illicit opioids would increase at the rate observed in preceding years, but would stabilize by 2020. The pessimistic scenario assumed that the opioid overdose crisis would not stabilize until 2025.

Assuming no change in the incidence of prescription opioid misuse (ie, constant incidence) from 2015 onward, opioid overdose deaths will not reach its peak value by 2025 (Figure 4A). If the incidence of prescription opioid misuse continues to decrease at the rate observed during 2008 to 2015 (ie, 7.5% per year), the number of overdose deaths would peak at 75 400 (95% UI, 61 900-90 600) in 2022 and remain relatively stable thereafter. The total number of overdose deaths between 2016 to 2025 would be 674 000 (95% UI, 562 600-793 700), a 3.8% decrease (2.0% decrease in deaths from illicit opioids and 10.7% decrease in deaths from prescription opioids) compared with the constant incidence scenario (Figure 4C and Table). Further decreasing the incidence of prescription opioid misuse at a 50% higher rate than the historical rate (ie, a 11.3% reduction per year) would decrease the total number of overdose deaths by 5.3% (a 2.8% and a 14.9% decrease in deaths from illicit and prescription opioids, respectively) compared with the circumstance of constant incidence. Under an extreme, hypothetical case of no incidence of prescription opioid misuse after 2015, the total number of overdose deaths would decrease by 17.3% compared with the circumstance of constant incidence. In all strategies, the number of deaths in 2025 would still remain higher than that in 2015. Similar results were observed in the sensitivity analysis that accounted for the missing populations in the NSDUH data (eAppendix 4, eTable 5, and eFigures 6 and 7 in the Supplement).

**Table.** Model-Projected Opioid Overdose Deaths Under 4 Interventions of Preventing Prescription Opioid Misuse, Each Defined by Their Projected Effect on the Incidence of Prescription Opioid Misuse

Incidence of Nonmedical Prescription Opioid Use, 2016-2025	No. of OD Deaths From Prescription or Illicit Opioids, 2016-2025 (% Change)^a^	No. of OD Deaths From Illicit Opioids, 2016-2025 (% Change)^a^	No. of OD Deaths From Prescription Opioids, 2016-2025 (% Change)^a^
Base-case scenario (opioid overdose crisis stabilizes by 2020)^b^			
No change since 2015, No.	700 400	557 140	143 260
Incidence decreases by 7.5% per year	674 030 (−3.8)	546 130 (−2.0)	127 900 (−10.7)
Incidence decreases by 11.3% per year	663 500 (−5.3)	541 600 (−2.8)	121 900 (−14.9)
No new incidence	579 170 (−17.3)	500 840 (−10.1)	78 320 (−45.3)
Pessimistic scenario (opioid overdose crisis stabilizes by 2025)^b^			
No change since 2015, No.	1 205 430	1 062 170	143 260^c^
Incidence decreases by 7.5% per year	1 168 720 (−3.0)	1 040 820 (−2.0)	127 900 (−10.7)^c^
Incidence decreases by 11.3% per year	1 154 010 (−4.3)	1 032 110 (−2.8)	121 900 (−14.9)^c^
No new incidence	1 036 460 (−14.0)	958 130 (−9.8)	78 320 (−45.3)^c^

Abbreviation: OD, overdose.

^a^ Change relative to the assumption of constant incidence (no change since 2015).

^b^ The base-case scenario assumed that the opioid overdose crisis will stabilize by 2020 (ie, the incidence of illicit opioids as the initiating opioid and the overdose mortality rate attributable to illicit opioids would increase at the rate observed in preceding years, but would stabilize by 2020). The pessimistic scenario assumed that the opioid overdose crisis would not stabilize until 2025.

^c^ Results of number of OD deaths from prescription opioids in the pessimistic scenario are the same as those in the base-case scenario, because the changes of assumptions in incidences and overdose mortality from illicit opioids would not affect the outcomes of the population using prescription opioids with and without opioid use disorder.

### Scenario Analysis of Outcomes Under Pessimistic Scenario

Under the pessimistic scenario (ie, illicit opioid lethality and incidence will stabilize by 2025), we projected that the number of opioid overdose deaths would continue to increase, reaching 198 700 (95% UI, 145 900-261 700) in 2025 (a 500% increase from 2015) (Figure 4B). We projected that 1.21 million individuals (95% UI, 0.97-1.47 million) would die from opioid overdose between 2016 and 2025, and 88% of those deaths would be attributable to illicit opioids. In this scenario, decreasing the incidence of prescription opioid misuse by prevention interventions to any level would not bend the curve of overdose deaths to a decreasing trend. Furthermore, these efforts only would have a modest effect on the cumulative number of overdose deaths during 2016 to 2025, ie, a 3.0% to 4.3% decrease compared with the circumstance of constant incidence (Figure 4D and Table).

### Model Calibration and Validation

Model-projected overdose deaths from prescription opioids and from illicit opioids closely replicated the outcomes reported by the CDC during 2002 to 2015 (Figure 2). Furthermore, the calibrated model projected the number of opioid overdose deaths for years 2016 and 2017 that closely matched with the reported deaths by the CDC^29^ (42 600 deaths [95% UI, 38 900-46 400] by model projection vs 42 247 by CDC in year 2016, 52 000 deaths [95% UI, 47 300-56 900] vs 49 068 in 2017). The model also closely replicated observed temporal trends of all other calibration targets from 2002 to 2015 (Figure 3).

## Discussion

Using a system dynamics model that closely replicates key trends in the opioid overdose epidemic, we found that the number of opioid overdose deaths in the United States is likely to continue to increase in the near term. We projected that the annual number of opioid overdose deaths will reach 81 700 in 2025, and from 2016 to 2025, 700 400 individuals could die from opioid overdose, with 80% of these deaths attributable to illicit opioids. Most important, we found that even substantial decreases in the incidence of prescription opioid misuse—that could be achieved, in theory, by highly successful prevention of prescription opioid misuse—would result only in a modest decrease of 3.8% to 5.3% in opioid-related overdose deaths during 2016 to 2025. While there is considerable uncertainty in the number of deaths by 2025, none of the studied interventions are projected to bring down the overdose deaths to current levels. Together, these findings highlight the limitations of prevention of prescription opioid misuse alone, and the need to use multiple policy levers simultaneously, ie, prevention, treatment, and harm reduction, to alter the projected course of the opioid overdose crisis in the coming years.

Our study also highlights the changing nature of the epidemic. The opioid crisis is expected to worsen in the next decade owing to multiple factors. First, the number of individuals using illicit opioids is expected to increase substantially. Second, unlike historical trends where prescription opioid use has served as a path to heroin use, more people are directly initiating opioid use with illicit opioids.^17^ Third, there has been a rapid increase in illicit opioid lethality, likely mainly driven by the infiltration of the heroin supply with the highly potent synthetic opioid fentanyl.^26,27^ However, patterns of accessibility and lethality of illicit opioids vary by region, with the infiltration of fentanyl in the South and West behind that in Northeast.^27^ In Massachusetts, for example, the number of opioid overdose deaths may have peaked in 2016,^28^ whereas the number continues to increase in most other states.

While our study’s conclusions regarding the inadequacy of the projected effect of lowering prescription opioid supply on opioid overdose deaths are similar to findings of a recent study by Pitt and colleagues,^30^ our model projected a higher number of opioid overdose deaths, with a total of 700 400 overdose deaths from 2016 to 2025 compared with 510 000 overdose deaths as estimated by Pitt and colleagues’ model.^30^ This difference may be due to our model structure, in which we explicitly considered the changing nature of the opioid overdose epidemic, including increasing trends over time, in the incidence and lethality of illicit opioid use. Our study further projected that the total number of opioid overdose deaths from 2016 to 2025 could reach as high as 1.21 million if the opioid overdose crisis does not stabilize soon.

In response to the growing burden of the opioid crisis and OUD, state and local governments have instituted several interventions aimed at preventing individuals from exposure to prescription opioids, including a recently proposed goal to lower opioid prescriptions by one-third in the coming 3 years.^31^ Our study does not devalue these efforts and it is possible that their effect could improve over time, which may ultimately yield a substantial benefit in the long term. However, given the large number of individuals who have already engaged in prescription opioid misuse or illicit opioid use, our study indicates that prevention efforts, in isolation, are unlikely to have the desired level of effect on opioid overdose deaths the near term.

Changing the course of the opioid crisis will require a multipronged approach. It could include implementation of screening for OUD in all relevant health care settings,^32^ improving access to medications for OUD such as methadone and buprenorphine,^33,34,35^ increasing OUD training programs at medical and nursing schools,^32,35^ improving access to harm-reduction services,^33^ and controlling the supply of illicit opioids. Implementation of these strategies will require health care professionals and communities to further overcome the stigma of opioid use and OUD, and to develop innovative point-of-care ways of delivering related services to those in need. Our model analysis that evaluates only the prevention of prescription opioids is a valuable first step; future analyses are needed to evaluate the effectiveness of a multipronged approach to lower opioid overdose deaths.

### Limitations

This study has several limitations. First, our model was calibrated to data sources, which could represent underestimates. Specifically, the NSDUH is conducted in the civilian, noninstitutionalized population excluding homeless and incarcerated populations. It also relies on respondents’ self-report of socially stigmatized behaviors such as heroin use. Furthermore, the percentage of individuals using illicit opioids as their first opioid of use was based on a survey from opioid users entering treatment programs. On the other hand, our sensitivity analysis showed that our study’s conclusions remain robust. Second, we made assumptions regarding the future trajectory of the opioid overdose epidemic. The fact that we could independently validate the model-projected number of opioid overdose deaths in 2016 and 2017 with CDC data provides confidence in the reliability of our findings. Furthermore, our primary finding, that interventions that focused on lowering prescription opioid misuse provide relatively modest public health benefits, was consistent across all scenarios tested. Third, we acknowledge that substantial heterogeneity may exist at state or local levels in regard to the burden and dynamics of the opioid epidemic,^36^ and thus the effectiveness of intervention policies could vary owing to local factors. Our study did not account for region-specific effectiveness of interventions owing to the lack of available data; however, our results highlight that even when the incidence of prescription opioid misuse can be effectively lowered, its effect on overdose deaths may be limited.

## Conclusions

We found that under current conditions the opioid overdose crisis is likely to substantially worsen and that interventions such as prescription drug monitoring programs are unlikely to lead to major decreases in the number of deaths from opioid overdose in the near future. Given these findings, policymakers will need to take a stronger and multipronged approach, such as improving access to treatment, expanding harm-reduction interventions, and lowering exposure to illicit opioids, to curb the trajectory of the opioid overdose epidemic in the United States.

## Notes

Supplement. **eAppendix 1.** Details of System Dynamics Model **eAppendix 2.** Model Parameter Estimation and Calibration **eAppendix 3.** Parameters for Model Projection **eAppendix 4.** Additional Sensitivity Analysis for NSDUH Estimates **eFigure 1.** Schematic of the System Dynamics Model of Nonmedical Opioid Use **eFigure 2.** Joinpoint Regression Analysis Based on NSDUH and CDC WONDER Data **eFigure 3.** Distribution of Calibrated Model Parameter Values Over Their Search Ranges **eFigure 4.** Changes to Model Parameters That Were Used in the Base Case and Pessimistic Projection Scenario **eFigure 5.** Scenarios of Projected Incidence of Prescription Opioid Misuse **eFigure 6.** Sensitivity Analysis: Temporal Trends in the Opioid Overdose Crisis With Model Re-Calibrated to the Adjusted Prevalence and Incidence Estimates From NSDUH Data **eFigure 7.** Sensitivity Analysis: Effects of Preventing New Cases of Prescription Opioid Misuse in the Pessimistic and Optimistic Scenarios With Model Recalibrated to the Adjusted Prevalence and Incidence Estimates From NSDUH Data **eTable 1.** Time-Dependent Structure of Model Parameters Informed by the Joinpoint Analysis **eTable 2.** Calibration Targets by Year From 2002-2015 **eTable 3.** Model Calibration Targets and Corresponding Model Outputs **eTable 4.** Estimates and Ranges of Calibrated Model Parameters **eTable 5.** Sensitivity Analysis: Projected Opioid Overdose Deaths With Model Recalibrated to the Adjusted Prevalence and Incidence Estimates From NSDUH Data **eReferences**
