# Supplementary material for: Prevention of Prescription Opioid Misuse and Projected Overdose Deaths in the United States
Source: JAMA Netw Open. 2019 Feb 1;2(2):e187621. doi: 10.1001/jamanetworkopen.2018.7621 (PMC6415966; doi:10.1001/jamanetworkopen.2018.7621)
Supplement: Supplement. — eAppendix 1. Details of System Dynamics Model eAppendix 2. Model Parameter Estimation and Calibration eAppendix 3. Parameters for Model Projection eAppendix 4. Additional Sensitivity Analysis for NSDUH Estimates eFigure 1. Schematic of the System Dynamics Model of Nonmedical Opioid Use eFigure 2. Joinpoint Regression Analysis Based on NSDUH and CDC WONDER Data eFigure 3. Distribution of Calibrated Model Parameter Values Over Their Search Ranges eFigure 4. Changes to Model Parameters That Were Used in the Base Case and Pessimistic Projection Scenario eFigure 5. Scenarios of Projected Incidence of Prescription Opioid Misuse eFigure 6. Sensitivity Analysis: Temporal Trends in the Opioid Overdose Crisis With Model Re-Calibrated to the Adjusted Prevalence and Incidence Estimates From NSDUH Data eFigure 7. Sensitivity Analysis: Effects of Preventing New Cases of Prescription Opioid Misuse in the Pessimistic and Optimistic Scenarios With Model Recalibrated to the Adjusted Prevalence and Incidence Estimates From NSDUH Data eTable 1. Time-Dependent Structure of Model Parameters Informed by the Joinpoint Analysis eTable 2. Calibration Targets by Year From 2002-2015 eTable 3. Model Calibration Targets and Corresponding Model Outputs eTable 4. Estimates and Ranges of Calibrated Model Parameters eTable 5. Sensitivity Analysis: Projected Opioid Overdose Deaths With Model Recalibrated to the Adjusted Prevalence and Incidence Estimates From NSDUH Data eReferences [file jamanetwopen-2-e187621-s001.pdf]

Supplementary Online Content

Chen Q, Larochelle MR, Weaver DT, et al. Prevention of prescription opioid misuse and projected overdose deaths in the United States. *JAMA Netw Open*. 2019;2(2):e187621. doi:10.1001/jamanetworkopen.2018.7621

eAppendix 1. Details of System Dynamics Model

eAppendix 2. Model Parameter Estimation and Calibration

eAppendix 3. Parameters for Model Projection

eAppendix 4. Additional Sensitivity Analysis for NSDUH Estimates

eFigure 1. Schematic of the System Dynamics Model of Nonmedical Opioid Use

eFigure 2. Joinpoint Regression Analysis Based on NSDUH and CDC WONDER Data

eFigure 3. Distribution of Calibrated Model Parameter Values Over Their Search Ranges

eFigure 4. Changes to Model Parameters That Were Used in the Base Case and Pessimistic Projection Scenario

eFigure 5. Scenarios of Projected Incidence of Prescription Opioid Misuse

eFigure 6. Sensitivity Analysis: Temporal Trends in the Opioid Overdose Crisis With Model Re-Calibrated to the Adjusted Prevalence and Incidence Estimates From NSDUH Data

eFigure 7. Sensitivity Analysis: Effects of Preventing New Cases of Prescription Opioid Misuse in the Pessimistic and Optimistic Scenarios With Model Recalibrated to the Adjusted Prevalence and Incidence Estimates From NSDUH Data

eTable 1. Time-Dependent Structure of Model Parameters Informed by the Joinpoint Analysis

eTable 2. Calibration Targets by Year From 2002-2015

eTable 3. Model Calibration Targets and Corresponding Model Outputs

eTable 4. Estimates and Ranges of Calibrated Model Parameters

eTable 5. Sensitivity Analysis: Projected Opioid Overdose Deaths With Model Recalibrated to the Adjusted Prevalence and Incidence Estimates From NSDUH Data

eReferences

© 2019 Chen Q et al. *JAMA Network Open*.

This supplementary material has been provided by the authors to give readers additional information about their work.

© 2019 Chen Q et al. *JAMA Network Open*.

eAppendix 1. Details of System Dynamics Model

We developed a system dynamics model (also known as *compartmental model*) (1), the ***O**pioid **P**olicy **M**odel* (*OPyM*), to simulate the opioid overdose crisis in the United States (US) from 2002–2025, and used it to assess the effects of reducing the incidence of nonmedical opioid analgesic use on the number of opioid overdose deaths.

We estimated the values of model parameters directly from data, where possible, including the National Survey on Drug Use and Health (NSDUH) (2), and the multiple cause of death data from the Centers for Disease Control and Prevention (CDC) (3). For parameters that could not be informed by available data sources, we fitted them using a model calibration approach. Further details of parameter estimation and calibration are described in **eAppendix 2**.

In this section, we describe the definition, structure, and evaluation of the system dynamic model in further detail, specifically:

1) Definition of compartments and the mathematical system dynamics model;
2) Formulation of time-dependent model parameters using joinpoint regression analysis;
3) Model evaluation accounting for uncertainty in input parameters.

A1.1 System Dynamics Model Structure

The system dynamics model consists of three compartments that represent the major subgroups of the population that use opioids (prescription and illicit) non-medically. The definitions of these population groups were adapted from the available information in the NSDUH survey data. In particular, we categorized the population with non-medical opioid use into the following three groups (**eFigure 1**):

1) People with *non-medical use of prescription opioids*, denoted by compartment (N). The corresponding selection criteria for this group in the NSDUH data include all the respondents who claimed to use opioids non-medically in the past year, did not claim heroin use in the past year, and did not meet opioid use disorder DSM-IV (Diagnostic and Statistical Manual of Mental Disorders 4th Edition) diagnosis criteria;
2) People with *prescription opioid use disorder (OUD)*, denoted by compartment (D). The corresponding selection criteria for this group in the NSDUH data include respondents who met DSM-IV criteria for opioid abuse or dependence and did not claim to use heroin in the past year;
3) People with *illicit opioid use*, denoted by compartment (I). The corresponding selection criteria for this group in the NSDUH data include respondents who claimed to use illicit heroin/fentanyl (regardless of the route of administration).
    a. It is worth noting that it is possible that an individual uses both prescription opioids and heroin at the same time in real-world situations. However, by our hierarchical definitions of compartments, if the person claimed to use heroin/fentanyl in the past-year, he/she is categorized into the illicit opioid use compartment (I).

© 2019 Chen Q et al. *JAMA Network Open*.

b. To keep the model parsimonious, we did not separate illicit opioid use without vs. with OUD. Separating it into different compartments would have increased the number of model parameters whose estimates are not readily available (e.g., transition rate from illicit opioid use to illicit OUD), without allowing us to query additional policy implications that were relevant the scope of this study.

Time progresses continuously in the model. As shown in **eFigure 1**, new individuals enter the model using either prescription opioids non-medically (compartment N) or illicit opioids (compartment I), and then transition through different compartments, i.e., progressing through different states of opioid use. From non-medical use of prescription opioids, individuals can develop a prescription OUD (compartment D). Individuals who used prescription opioids, with or without prescription OUD (compartments N and D), can transition to illicit opioid use (compartment I). Individuals were subject to opioid overdose death with mortality rates dependent on their compartment. In addition, individuals could transition out of the model when they either stopped using opioids or died from other (i.e., non-opioid-related) causes.

Mathematically, we used $N(t), D(t), I(t)$ to represent the prevalence for each compartment at time t, and the system dynamics were represented by the following ordinary differential equations:

$$\frac{dN(t)}{dt} = \lambda_N(t) - (\mu_N + m_N + p_{ND} + p_{NI}) \cdot N(t),$$

$$\frac{dD(t)}{dt} = p_{ND} \cdot N(t) - \big(\mu_D(t) + m_D(t) + p_{DI}(t)\big) \cdot D(t),$$

$$\frac{dI(t)}{dt} = \lambda_I(t) + p_{DI}(t) \cdot D(t) + p_{NI} \cdot N(t) - \big(\mu_I + m_I(t)\big) \cdot I(t),$$

with initial conditions $\big(N(t_0), D(t_0), I(t_0)\big) = (N_0, D_0, I_0)$. Model variables are described as follows:

• $N(t), D(t), I(t)$:	The prevalence of non-medical use of prescription opioids (compartment N), prescription OUD (compartment D), and illicit opioid use (compartment I), respectively, at time t.
• N_0, D_0, I_0:	Initial value for each compartment at time t_0, i.e., year 2002.
• $\lambda_N(t)$ and $\lambda_I(t)$:	Annual incidence of non-medical use of prescription opioids and of illicit opioid use from other sources (i.e., not from prescription opioids), respectively, at time *t.* The model differentiates two cases of incident illicit opioid use: those who progress to illicit opioid use from prescription opioid use and those who initiate opioid use with illicit opioids; $\lambda_I(t)$ refers to the latter case.

• $p_{ND}, p_{DI}(t), p_{NI}$:	Annual transition rate from compartments *N* to *D*, *D* to *I*, and *N* to *I*, respectively, at time t.
• $m_N, m_D(t), m_I(t)$:	Overdose mortality rate from compartments *N*, *D*, and *I*, respectively, at time *t*.
• $\mu_N, \mu_D(t), \mu_I$:	Exit rate from compartments *N*, *D*, and *I*, respectively, at time *t*.

The system dynamics model was implemented in R programming language (Version 3.4.0) using "deSolve" package to solve the ordinary differential equations.

For some model parameters, we considered time-dependent structure in their values, because a constant value over time may not be sufficient to capture the highly dynamic trends in the current opioid epidemic, such as rapidly increasing overdose deaths in the past few years, and the increasing proportion of opioid initiates from illicit opioids. Next, we describe the formulation of time-dependent parameters.

A1.2 Joinpoint Model for Time-Dependent Parameters

To model the time-dependent structure of model parameters, we applied joinpoint regression analysis (4, 5), which is commonly used to describe trends over time and identify changes in a trend at certain time points. A joinpoint regression model for parameter $\beta(t)$ consists of the following components:

1) β_0, the baseline value at the initial time point t_0;
2) α_1, annual percentage of change (APC) before the joinpoint;
3) τ, a joinpoint time indicating a significant change of APC from time τ onwards (i.e., an inflection point of trend at time τ); and
4) α_2, APC after the joinpoint τ.

Mathematically, time-dependent parameter $\beta(t)$ is determined as follows:

$$\beta(t) = \begin{cases} \beta_0 \cdot (1+\alpha_1)^t & \text{if } t_0 < t < \tau, \\ \beta_0 \cdot (1+\alpha_1)^\tau \cdot (1+\alpha_2)^{t-\tau} & \text{if } t \geq \tau. \end{cases}$$

If there is no joinpoint τ specified, $\beta(t)$ increases/decreases with APC α_1 indefinitely since time t_0. Joinpoint regression can also be generalized to include more than one joinpoint.

For our system dynamics model, we first estimated the annual incidence of prescription opioid misuse from 2002–2014 using the NSDUH data (see A2.1 for estimation details), and then applied the joinpoint regression analysis on the annual incidence to obtain the joinpoint equation for $\lambda_N(t)$ (**eFigure 2A, eTable 1**). Instead of directly using the raw annual incidence estimates from NSDUH data, we used the joinpoint regression equation in our system dynamics model to avoid unsmooth variations in the incidence values across years.

© 2019 Chen Q et al. *JAMA Network Open*.

To estimate time-dependent values of the overdose mortality rates, $m_D(t)$ and $m_I(t)$, we performed the joinpoint regression analysis on the multiple cause of death data from the CDC Wide-Ranging Online Data for Epidemiologic Research (WONDER) for 2002–2015, and identified inflection points in the numbers of annual overdose deaths (**Figures 2B and 2C**). In particular, we incorporated the joinpoint year of 2011 for overdose mortality rate from illicit opioids $m_I(t)$, and a joinpoint year of 2007 for overdose mortality rate from prescription OUD $m_D(t)$ (**eTable 1**). However, the baseline value, α_1, and α_2 obtained from the joinpoint regression equations cannot be directly used for parameters $m_D(t)$ and $m_I(t)$, because the observed data from CDC WONDER were the counts of overdose deaths, whereas the parameters $m_D(t)$ and $m_I(t)$ represented the transition rates per year (i.e., annual risks) of overdose deaths. Thus, in the joinpoint regression equations of the mortality rate parameters $m_D(t)$ and $m_I(t)$, we implemented the joinpoint years informed by the joinpoint analysis of CDC WONDER data, but determined the baseline value, α_1, and α_2 via model calibration (see **eAppendix 2**).

For the incident illicit opioid use ($\lambda_I(t)$), transition rate from prescription OUD to illicit opioid use ($p_{DI}(t)$), and exit rate from compartments D ($\mu_D(t)$), we considered a simpler time-dependent model structure without a joinpoint, i.e., a simple growth models with fixed α_1, as we found that such structures provided better fit to the observed data (i.e., model calibration) compared with assuming constant transition rates. Due to lack of information for inferring the joinpoint time, we kept the time-dependent model simplistic, and their baseline values and APC α_1 were determined through model calibration (**eAppendix 2**).

© 2019 Chen Q et al. *JAMA Network Open*.

eAppendix 2. Model Parameter Estimation and Calibration

In this section, we describe the estimation and calibration of parameter values in our system dynamics model. To account for model uncertainty, we also describe the sampling distributions of input parameter values. Different input parameter values were sampled for each model evaluation, which was repeated by 1000 times. From the 1000 replications of model evaluations, we presented the mean results (i.e., the average value of model outputs), and presented the 95% uncertainty interval using the 2.5th–97.5th percentile range of model outputs.

A2.1. Parameter Estimation

For the following parameters, we estimated their values directly from the NSDUH data:

1) Initial prevalence values, N_0, D_0, and I_0 for each compartment at year 2002. We first performed joinpoint regression analysis on the prevalence of each compartment estimated from the NSDUH data. The fitted joinpoint regression equations provided the estimates of the expected counts at each year, and we used the estimates at the year 2002 for the initial prevalence values. To account for uncertainty in those values, we used the standard error estimates from the NSDUH data and sampled the initial prevalence values assuming normal distribution as follows:

- Initial value of compartment N: N_0 with mean 10,029,859 and standard deviation 329,807;
- Initial value of compartment D: D_0 with mean 1,369,218 and standard deviation 116,347;
- Initial value of compartment I: I_0 with mean 328,731 and standard deviation 60,446.

2) Incidence of prescription opioid misuse $\lambda_N(t)$ at time t. We first estimated the annual incidence of non-medical use of prescription opioids using the NSDUH survey data from 2003-2014. To capture the annual incidences for the full year, we calculated the incidence for each calendar year using the survey data from the following year. For example, incidence estimate for 2002 was calculated from survey data in 2003. Specifically, we queried for respondents of the survey in 2003 who claimed having initiated opioids in the previous year, i.e., in 2002. This way, it allowed us to capture an entire 12-month period for incidence (otherwise, using the 2002 survey data would underestimate the incidence for the 2002 year, because the survey could take place anywhere during the year, leaving the new incidences after the survey excluded). The survey data from 2015 was not used to calculate the 2014 incidence because the survey questions changed and the variables available were no longer synonymous. We used survey weights to estimate the incidence of non-medical use of prescription opioids for the entire US population.

As indicated in Section A1.2, we then conducted the joinpoint analysis on the above estimates of annual incidence of prescription opioid misuse from the NSDUH data, which provided the estimates and the standard error of baseline value, α_1 and α_2 of the joinpoint regression equation (see **eTable 1**). We sampled these three values from a

© 2019 Chen Q et al. *JAMA Network Open*.

normal distribution based on the estimates of mean and standard deviation to determine the equation of $\lambda_N(t)$.

A2.2. Model Calibration

Because some model parameters are not directly observable from existing datasets, we used calibration methods (6-8) to estimate their values and ranges such that the model outcomes would closely match existing data. In this section, we describe the calibration parameters, targets, goodness-of-fit measure, search algorithm, and calibration results.

Calibration parameters

A total of 17 model parameters were determined by the model calibration, which are defined below:

- m_N: overdose mortality from compartment N (1 parameter);
- $m_D(t)$ and $m_I(t)$: joinpoint model for overdose mortality (baseline, α_1, and α_2) from compartment D and I, respectively (6 parameters);
- p_{ND} and p_{NI}: progression rate from compartment N to D, and from N to I, respectively (2 parameters);
- $p_{DI}(t)$: joinpoint model for progression rate from D to I: baseline and α_1 (2 parameters);
- $\lambda_I(t)$: joinpoint model for incidences of initiating illicit opioids: baseline and α_1 (2 parameters);
- μ_N and μ_I: exit rates for compartment N and I, respectively (2 parameters);
- $\mu_D(t)$: joinpoint model for exit rate for compartment D: baseline and α_1 (2 parameters).

Calibration targets

We compared the following model-predicted outcomes with observed data: prevalence of prescription opioid non-medical use, OUD, and illicit opioid use based on NSDUH data; (2) the number of overdoses from all opioids and illicit opioid use from on CDC WONDER data; (3) and the percentage of illicit opioid among initiating opioids based on a published study (**eTable 2**) (9).

Goodness-of-fit metric

For any given set of model parameters, we assessed how well the model was fitted to the calibration targets. That is, we compared the model outputs O_i with corresponding calibration targets E_i (**eTable 3**), and calculated the goodness-of-fit measure. For each calibration target, model error is defined by the sum of squared errors between model outputs and calibration targets over all years, and then normalized by the initial value of this target to preserve comparable magnitude across different calibration targets. The overall goodness-of-fit measure, the *total error*, was defined as the summation of model errors over all calibration targets (with equal weights):

© 2019 Chen Q et al. *JAMA Network Open*.

$$\text{total error} = \sum_{i} \sum_{t=t_1}^{t_n} \left(\frac{E_i(t) - O_i(t)}{E_i(t_1)} \times 100 \right)^2.$$

Calibration procedure

To calibrate the model, we used a directed search method to identify the sets of parameters that lead to “good” model fits. An optimization algorithm was applied to iteratively search for the parameter sets with the lowest possible total model errors. We systematically tested multiple optimization functions in R (L-BFGS algorithm with package “lbfgsb3”, Nelder-Mead algorithm with package “dfoptim”, Differential Evolution Optimization with package “DEoptim”, and Generalized Simulated Annealing with package “GenSA”), and selected package “GenSA”, which consistently outperformed other algorithms with good solution qualities within reasonable computation time.

Search for the “optimal” values of calibration parameters was bounded within the pre-determined range for each parameter. Because the search results from the optimization function (i.e., the calibration results) depend on the starting values of the search, we sampled the starting values for all parameters uniformly within their pre-determined ranges using *Latin hypercube sampling* approach and repeated the directed search for 1,000 replications. The parameter ranges were initially determined based on expert opinions and set conservatively wide.

For some parameters, we observed that the calibrated values were concentrated at the lower or upper bounds of their range. When this happened, we expanded the ranges and repeated the calibration process. We repeated this process until we observed that all calibrated parameters represented a “full” distribution of values (not a “truncated” distribution) over their search ranges. In addition, we made sure that the total calibration errors were reduced after each iteration of adjusting the search ranges. In this way, we believe that the search has adequately explored the promising areas with “good” values of model parameters that result in good model fitting.

Calibration results

The optimal solutions identified from 1,000 replications of model calibrations were collected and considered as the set of calibrated model parameters. To account for uncertainty in model input parameters, we did not average these parameter values into aggregated estimates for the model evaluation; instead, we evaluated the model outcomes using each of the 1,000 parameter sets and estimated 95% uncertainty intervals from the 1,000 replications of model evaluations. **eFigure 3** shows the distribution of calibrated parameter values over the search ranges for the calibration, and **eTable 4** summarizes values of the calibrated parameters. Model outcomes with calibrated parameters and comparisons with calibration targets can be found in Figures 2 and 3 of the main manuscript.

© 2019 Chen Q et al. *JAMA Network Open*.

eAppendix 3. Parameters for Model Projection

A3.1. Parameters for Projection Scenarios

To project model outcomes after year 2015, we considered three projection scenarios where the overdose mortality $m_I(t)$ and annual incidences $\lambda_I(t)$ of illicit opioid use will continue to increase after 2015 but stabilize at different time points in future. In particular, we simulated:

1) a *base-case scenario,* which assumes that the overdose mortality rate (i.e., lethality) of illicit opioids and the annual rate of incident illicit opioid use would stabilize by 2020; and
2) a *pessimistic scenario* which assumes that both rates would stabilize by 2025.

To implement such a structure, for any given APC value (i.e., α_2 for $m_I(t)$ and α_1 for $\lambda_I(t)$ that was obtained from model calibration), we decreased the value by a fixed amount every year after 2015 such that the APC decreases to 0 at the targeted stabilizing year (i.e., the year 2020 and 2025 in the base-case and pessimistic scenario, respectively); and the values of $m_I(t)$ and $\lambda_I(t)$ remain constant afterwards. **eFigure 4** illustrates the time-dependent structure of these two parameters for the base-case projection scenario.

A3.2 Simulated Strategies to Reduce the Incidence of Non-Medical Prescription Opioid Use

To evaluate the impact of reducing the incidence of non-medical opioid analgesic use on the number of opioid overdose deaths, we simulated the following four incidence trends representing different effects of strategies to reduce the incidence of non-medical prescription opioid use: **(1)** no change in the annual incidence of prescription opioid misuse from 2015 onward; **(2)** decreasing incidence of prescription opioid misuse from 2016–2025, at a rate of 7.5% per year as observed between 2008–2015 (i.e., α_2 in the joinpoint model for $\lambda_N(t)$ before 2015), which may be achieved by continued implementation and success of prevention programs; **(3)** decreasing incidence of prescription opioid misuse at a rate that is 50% faster than strategy 2 (i.e., a 11.3% decrease per year), which assumes greater success of prevention programs; and **(4)** a hypothetical setting of no new incidence of prescription opioid misuse after 2015, which was included to assess the maximum possible benefits of prevention interventions under ideal conditions. **eFigure 5** illustrates the retrospective incidence data prior to 2015 estimated from the NSDUH data, and four incidence trends from 2016 onwards that correspond with the above four prevention strategies (shaded areas represent uncertainty intervals).

© 2019 Chen Q et al. *JAMA Network Open*.

eAppendix 4. Additional Sensitivity Analysis for NSDUH Estimates

One of our model limitations is that we used NSDUH data for model calibration and parameterization, which does not capture homeless and incarcerated population and is based on self-reported outcomes. Thus, the NSDUH data could represent underestimates of the actual epidemic situation. Since no other data are readily available for such underrepresented population at national level, we conducted sensitivity analysis on our model parameters estimated from NSDUH data to assess the impact of the underestimates on our model results.

To determine the possible variability to the estimates obtained from NSDUH data and to adjust the parameter values used in our analysis, we searched the literature to compare the differences between the estimates from NSDUH and other independent data sources. We found that the NSUDH data estimated the prevalence of opioid abuse and dependence in Massachusetts to be 1.17% between 2009-2011 (10), while the estimate was 2.72-2.87% in 2011-2012 based on the Chapter 55 dataset that linked the Massachusetts All-Payer Claims Database and other state databases (11). Therefore, we doubled our estimates of prevalence for each compartment and incidence of prescription opioid non-medical use from NSDUH data as an approximation for the adjustment. Then we re-calibrated our model to the adjusted prevalence and incidence estimates (**eFigure 6**) and repeated our analysis.

We found that, with the doubled prevalence and incidence estimates, the projected number of overdose deaths in the base case was increased by only 5.5% (739,100 compared with 700,400 in the base case results, **eTable 5, eFigure 7**). Such a small change is mostly because the model was re-calibrated to the overdose deaths data from CDC WONDER. Furthermore, the relative reduction in cumulative overdose deaths from 2015-2025 attributable to reducing incidence of prescription opioid misuse became 3.7%-5.2% (**eTable 5**), which is comparable to the 3.8%-5.3% reduction in the base case results. We acknowledge that our adjustment to the NSDUH estimates is not exact, but this sensitivity analysis demonstrated that our model results are robust, and the limitation of underestimation by the NSDUH data would not substantially change our conclusions.

© 2019 Chen Q et al. *JAMA Network Open*.

eFigure 1. Schematic of the System Dynamics Model of Nonmedical Opioid Use

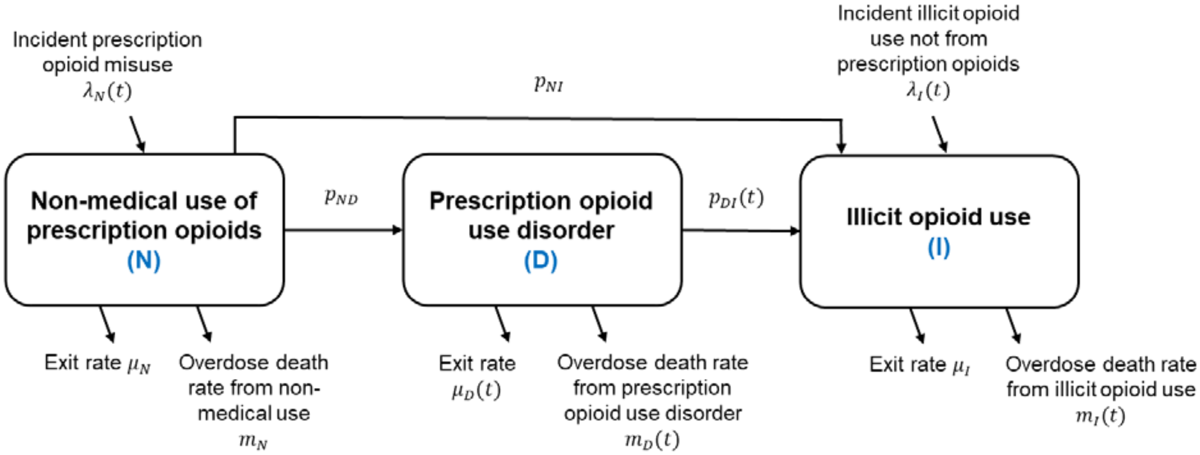

© 2019 Chen Q et al. *JAMA Network Open*.

eFigure 2. Joinpoint Regression Analysis Based on NSDUH and CDC WONDER Data

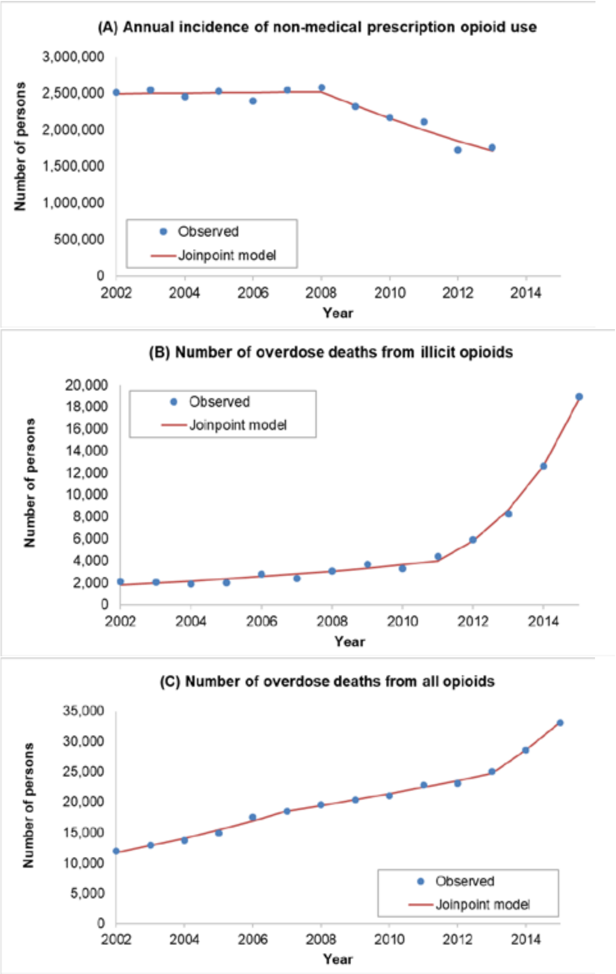

(A) Annual incidences of non-medical prescription opioid use, (B) number of overdose deaths from illicit opioids, (C) number of overdose deaths from all opioids. Point estimates represent data from NSDUH and CDC WONDER, and the lines represent the fitted joinpoint regression model.

© 2019 Chen Q et al. *JAMA Network Open*.

eFigure 3. Distribution of Calibrated Model Parameter Values Over Their Search Ranges

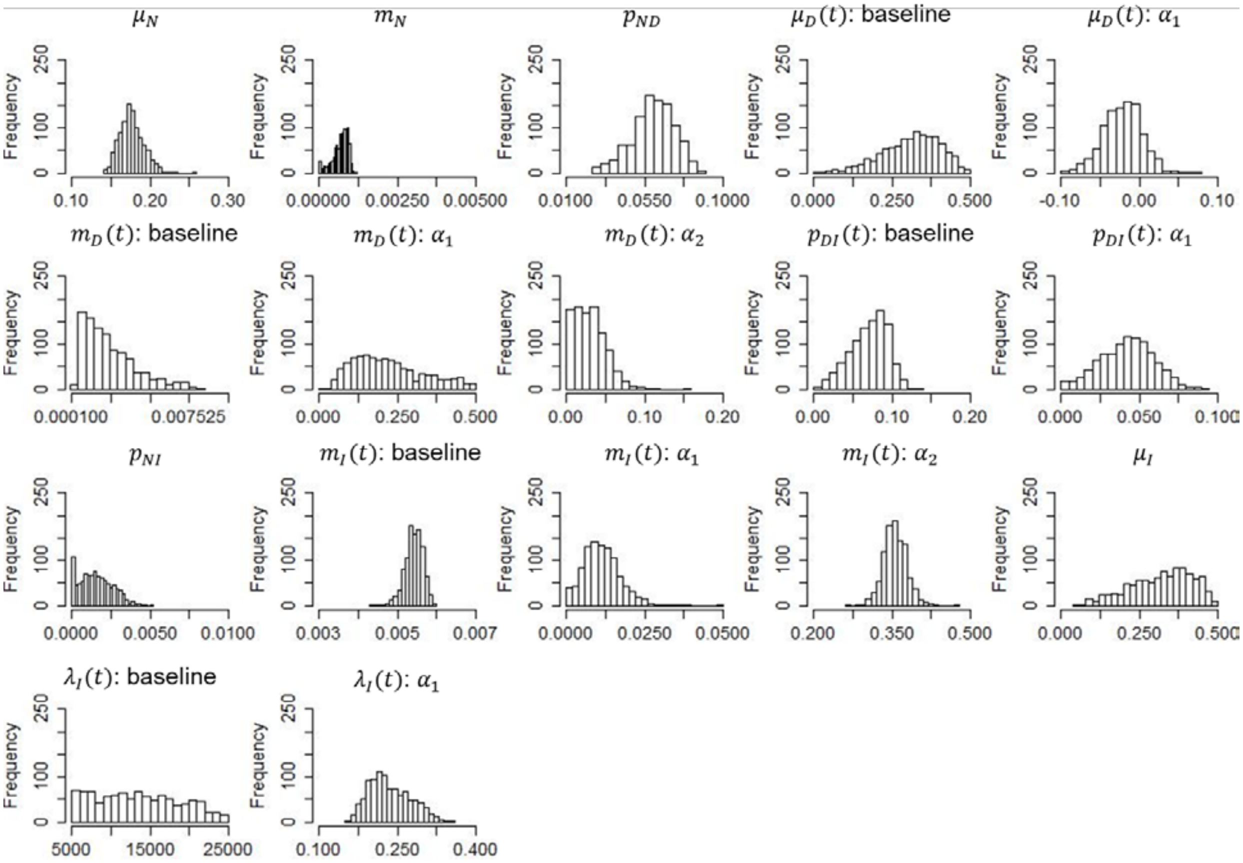

© 2019 Chen Q et al. *JAMA Network Open*.

eFigure 4. Changes to Model Parameters That Were Used in the Base Case and Pessimistic Projection Scenario

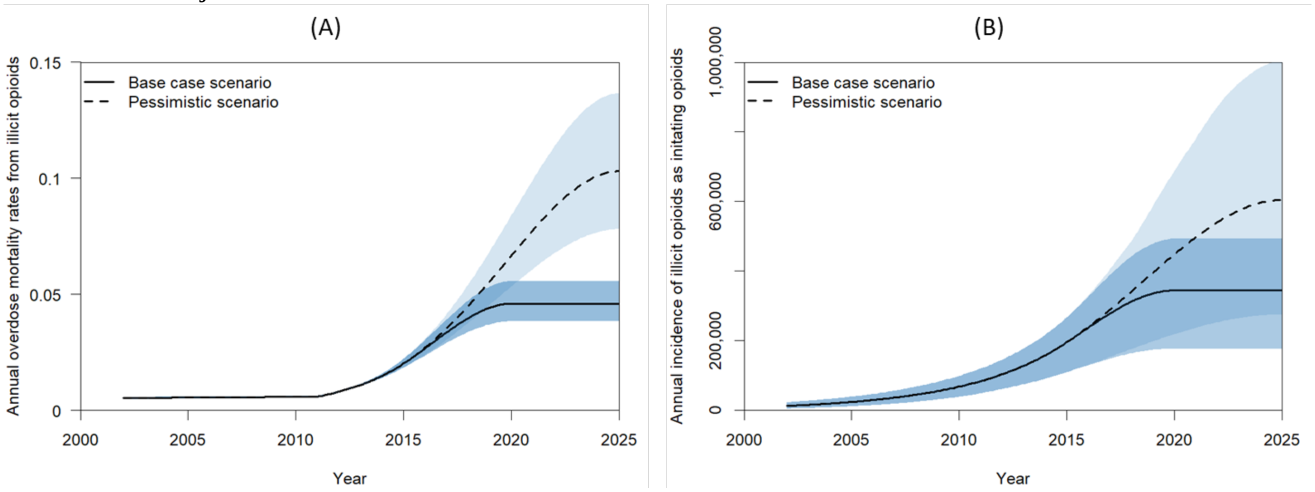

(A) Overdose mortality rates from illicit opioids $m_I(t)$, (B) annual incidence of illicit opioid use $\lambda_I(t)$. The solid and dashed lines represent the average values, and the band shows the 95% uncertainty interval based on the 1000 calibrated parameter values.

© 2019 Chen Q et al. *JAMA Network Open*.

eFigure 5. Scenarios of Projected Incidence of Prescription Opioid Misuse

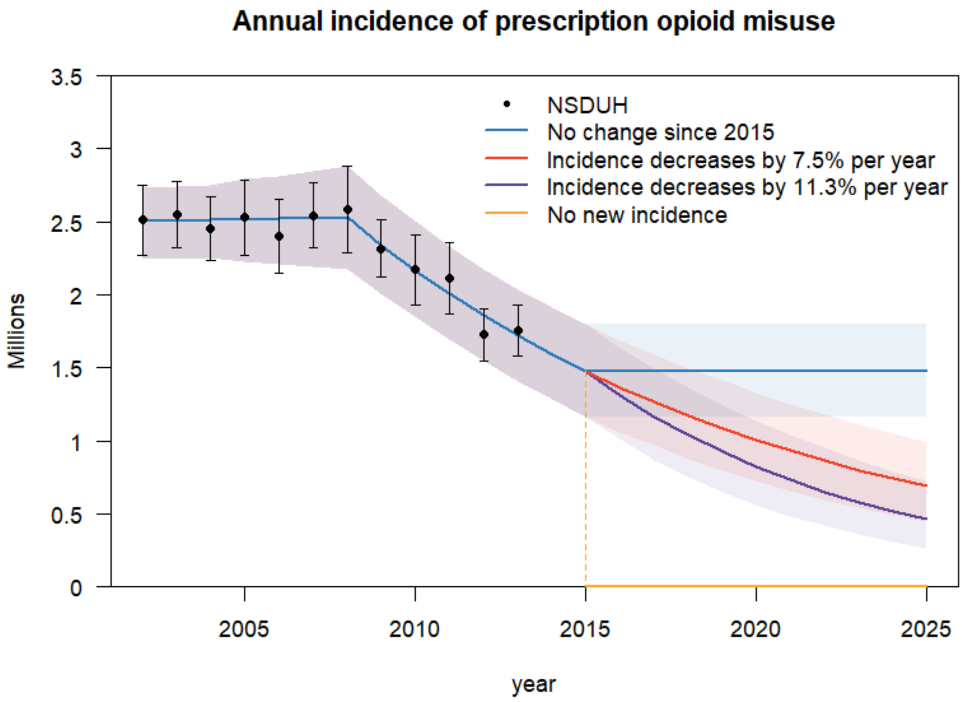

© 2019 Chen Q et al. *JAMA Network Open*.

eFigure 6. Sensitivity Analysis: Temporal Trends in the Opioid Overdose Crisis With Model Re-Calibrated to the Adjusted Prevalence and Incidence Estimates From NSDUH Data

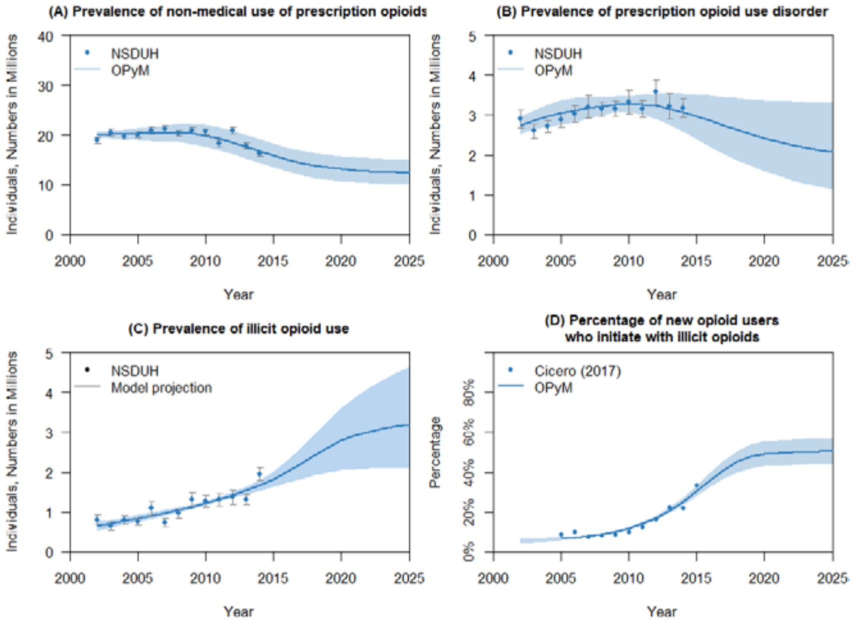

Lines represent the average of 1000 outcomes from the model. Error bars represent 95% confidence intervals of the observed outcomes from the NSDUH data, and blue shaded regions represent the bootstrapped 95% uncertainty intervals of the model outcomes.

Abbreviations: NSDUH, National Survey on Drug Use and Health. Cicero (2017) (9) refers to the source of calibration targets.

© 2019 Chen Q et al. *JAMA Network Open*.

eFigure 7. Sensitivity Analysis: Effects of Preventing New Cases of Prescription Opioid Misuse in the Pessimistic and Optimistic Scenarios With Model Recalibrated to the Adjusted Prevalence and Incidence Estimates From NSDUH Data

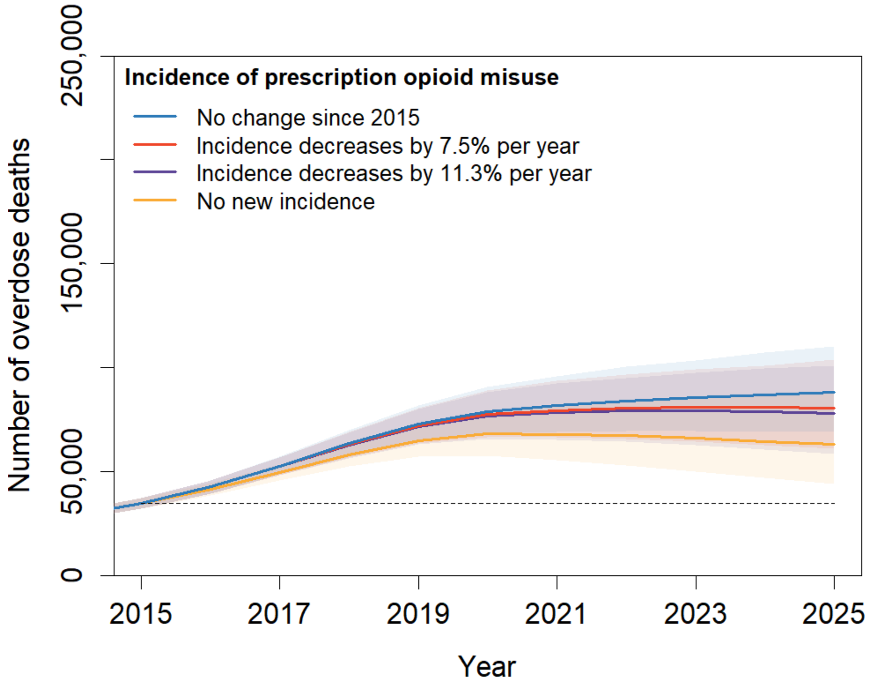

© 2019 Chen Q et al. *JAMA Network Open*.

eTable 1. Time-Dependent Structure of Model Parameters Informed by the Joinpoint Analysis

Parameter	Baseline value	α_1	Joinpoint τ	α_2	Data source
$\lambda_N(t)$	2,496,835 (SE: 124,146.9)	0.16% (SE for slope* = 0.0086)	2008	-7.47% (SE for slope* = 0.011)	Annual incidence of prescription opioid non-medical use from NSDUH data (2), defined as first-time non-medical use of prescription opioids in the previous year, from 2002 to 2014 (**eFigure 2A**).
$m_I(t)$	-	-	2011	-	Anuual number of overdose deaths from illicit opioids based on CDC WONDER(3) data from 2002 to 2015 (**eFigure 2B**).
$m_D(t)$	-	-	2007	-	Annual number of overdose deaths from all opioids excluding illicit opioids†, based on CDC WONDER (3) data from 2002 to 2015 (**eFigure 2C**).

* The joinpoint analysis provides SE for the slope, where APC = exp(slope)-1. Accordingly, slope for α_1 is 0.00165, and slope for α_2 is -0.0776.

† No time-dependent structure was assumed for m_N (the overdose mortality rate from compartment N). Considering the population without the diagnosis of opioid use disorder in compartment N, the rate is expected to be low and the temporal trend is not needed. For model simplicity, we considered m_N as a constant parameter with respect to time. And thus, we assumed that the inflection point of the trends in overdose deaths from all opioids at year 2007 was attributed to the change in overdose mortality from compartment D, i.e., $m_D(t)$.

SE, standard error; NSDUH, National Survey on Drug Use and Health; CDC, Centers for Disease Control and Prevention; WONDER, Wide-Ranging Online Data for Epidemiologic Research.

© 2019 Chen Q et al. *JAMA Network Open*.

eTable 2. Calibration Targets by Year From 2002-2015

Year	Prevalence of non-medical use of prescription opioids (compartment N)	Prevalence of prescription OUD (compartment D)	Prevalence of illicit opioid use (compartment I)	Number of overdose deaths from all opioids	Number of overdose deaths from illicit opioids	Percentage of illicit opioid among initiating opioids (9)
2002	9,451,560	1,453,161	403,624	11,919	2,089	-
2003	10,286,778	1,299,348	328,438	12,938	2,080	-
2004	9,853,595	1,354,599	404,359	13,753	1,881	-
2005	10,030,636	1,439,493	380,504	14,918	2,009	8.7%
2006	10,425,880	1,518,935	546,431	17,542	2,772	10.0%
2007	10,596,588	1,603,280	369,979	18,510	2,408	7.7%
2008	10,155,192	1,575,440	485,425	19,582	3,041	8.3%
2009	10,452,059	1,579,737	653,461	20,421	3,656	8.8%
2010	10,324,665	1,661,686	635,885	21,088	3,294	10.3%
2011	9,134,108	1,575,867	651,817	22,782	4,397	12.8%
2012	10,415,868	1,788,298	683,166	23,166	5,925	16.5%
2013	8,933,049	1,614,243	656,118	25,051	8,257	22.3%
2014	8,096,621	1,590,185	979,016	28,645	12,644	22.0%
2015	---*	---*	841,308	33,090	18,913	33.3%

* Estimates for year 2015 from NSDUH data were not used due to major changes in survey questions that year.

© 2019 Chen Q et al. *JAMA Network Open*.

eTable 3. Model Calibration Targets and Corresponding Model Outputs

**Index (i)**	**Calibration target (E_i)**	**Model output (O_i)**
**1**	Prevalence of non-medical use of prescription opioids	$N(t)$
**2**	Prevalence of prescription OUD	$D(t)$
**3**	Prevalence of illicit opioid use	$I(t)$
**4**	Overdose death from illicit opioid use	$m_I(t)I(t)$
**5**	Overdose death from all opioids (from all compartments)	$m_N N(t) + m_D(t)D(t) + m_I(t)I(t)$
**6**	Percentage of illicit opioid use at initial regular use of opioid	$\frac{\lambda_I(t) + N(t)p_{NI}}{N(t)p_{ND}(t) + \lambda_I(t) + N(t)p_{NI}}$

© 2019 Chen Q et al. *JAMA Network Open*.

eTable 4. Estimates and Ranges of Calibrated Model Parameters

Parameter	Value (SD)	Interquartile Range
μ_N	0.177 (0.0151)	0.166 - 0.186
m_N	0.001 (0.0002)	0.001 - 0.001
p_{ND}	0.06 (0.012)	0.053 - 0.069
$\mu_D(t)$: baseline	0.311 (0.092)	0.249 - 0.38
$\mu_D(t)$: α_1	-0.021 (0.0254)	-0.038 - -0.005
$m_D(t)$: baseline	0.003 (0.0016)	0.001 - 0.003
$m_D(t)$: α_1	0.224 (0.113)	0.134 - 0.292
$m_D(t)$: α_2	0.03 (0.021)	0.014 - 0.043
$p_{DI}(t)$: baseline	0.071 (0.0236)	0.054 - 0.088
$p_{DI}(t)$: α_1	0.042 (0.0172)	0.03 - 0.054
p_{NI}	0.002 (0.001)	0.001 - 0.002
$m_I(t)$: baseline	0.005 (0.0002)	0.005 - 0.006
$m_I(t)$: α_1	0.011 (0.0061)	0.007 - 0.015
$m_I(t)$: α_2	0.356 (0.0227)	0.342 - 0.371
μ_I	0.319 (0.0992)	0.248 - 0.395
$\lambda_I(t)$: baseline	13489 (5347)	8956 - 17608
$\lambda_I(t)$: α_1	0.235 (0.0399)	0.205 - 0.265

© 2019 Chen Q et al. *JAMA Network Open*.

eTable 5. Sensitivity Analysis: Projected Opioid Overdose Deaths With Model Recalibrated to the Adjusted Prevalence and Incidence Estimates From NSDUH Data

**Incidence of non-medical prescription opioid use, 2016–2025**	**Number of OD deaths from prescription or illicit opioids, 2016–2025 (% change)**		**Number of OD deaths from illicit opioids, 2016–2025 (% change)**		**Number of OD deaths from prescription opioids, 2016–2025 (% change)**	
**Base-case scenario (opioid overdose crisis stabilizes by 2020)**						
No change since 2015	739,090		593,730		145,360	
Incidence decreases by 7.5% per year	711,870	(-3.7%)	581,930	(-2%)	129,940	(-10.6%)
Incidence decreases by 11.3% per year	700,980	(-5.2%)	577,070	(-2.8%)	123,920	(-14.7%)
No new incidence	613,220	(-17%)	533,040	(-10.2%)	80,190	(-44.8%)
**Pessimistic scenario (opioid overdose crisis stabilizes by 2025)**						
No change since 2015	1,350,680		1,205,320		145,360	
Incidence decreases by 7.5% per year	1,311,760	(-2.9%)	1,181,820	(-1.9%)	129,940	(-10.6%)
Incidence decreases by 11.3% per year	1,296,130	(-4%)	1,172,210	(-2.7%)	123,920	(-14.7%)
No new incidence	1,170,110	(-13.4%)	1,089,920	(-9.6%)	80,190	(-44.8%)

© 2019 Chen Q et al. *JAMA Network Open*.

eReferences

1. Homer JB, Hirsch GB. System dynamics modeling for public health: background and opportunities. American journal of public health. 2006;96(3):452-8.
2. United States Department of Health and Human Services, Substance Abuse and Mental Health Services Administration, Center for Behavioral Health Statistics and Quality. National survey on drug use and health. Ann Arbor, MI: Inter-university Consortium for Political and Social Research; 2015.
3. Centers for Disease Control and Prevention, National Center for Health Statistics. Multiple cause of death 1999-2016 on CDC WONDER online database. http://wonder.cdc.gov/mcd-icd10.html; 2017.
4. Kim HJ, Fay MP, Feuer EJ, Midthune DN. Permutation tests for joinpoint regression with applications to cancer rates. Statistics in medicine. 2000;19(3):335-51.
5. Joinpoint Regression Program, Version 4.6.0.0 - April 2018. Statistical Methodology and Applications Branch, Surveillance Research Program, National Cancer Institute.
6. Stout NK, Knudsen AB, Kong CYJ, McMahon PM, Gazelle GS. Calibration methods used in cancer simulation models and suggested reporting guidelines. Pharmacoeconomics. 2009;27(7):533.
7. Kong CY, McMahon PM, Gazelle GS. Calibration of disease simulation model using an engineering approach. Value in Health. 2009;12(4):521-9.
8. Taylor DC, Pawar V, Kruzikas D, Gilmore KE, Pandya A, Iskandar R, et al. Methods of model calibration: observations from a mathematical model of cervical cancer. PharmacoEconomics. 2010;28(11):995-1000.
9. Cicero TJ, Ellis MS, Kasper ZA. Increased use of heroin as an initiating opioid of abuse. Addictive behaviors. 2017;74:63-6.
10. Jones CM, Campopiano M, Baldwin G, McCance-Katz E. National and state treatment need and capacity for opioid agonist medication-assisted treatment. American journal of public health. 2015;105(8):e55-e63.
11. Barocas JA, White LF, Wang J, Walley AY, LaRochelle MR, Bernson D, et al. Estimated Prevalence of Opioid Use Disorder in Massachusetts, 2011–2015: A Capture–Recapture Analysis. American Journal of Public Health. 2018(0):e1-e7.

© 2019 Chen Q et al. *JAMA Network Open*.
